# Psychosocial and energetic factors on human female pubertal timing: a systematized review

**DOI:** 10.1017/ehs.2022.24

**Published:** 2022-06-09

**Authors:** Delaney J. Glass, Joy T. Geerkens, Melanie A. Martin

**Affiliations:** 1 University of Washington, Department of Anthropology, Seattle, Washington, USA; 2 Brunel University, Department of Life Sciences, London, UK

**Keywords:** Puberty, stress, child growth, evolution

## Abstract

Childhood psychosocial stressors have been proposed to favour fast life history strategies promoting earlier puberty in females. However, studies demonstrating this association often do not elucidate causal mechanisms, nor account for greater childhood energetic availability – also known to promote rapid growth and earlier puberty. To assess the extent to which such confounding has been considered, we conducted a systematized review to identify studies examining measures of both prepubertal growth (e.g. weight, height) and psychosocial stressors (e.g. adversity, father absence) in relation to female pubertal timing. A total of 1069 non-duplicated studies were identified across five databases. Twenty studies met selection criteria for critical review following independent screening of titles, abstracts and manuscripts. Within these studies, measures indicative of rapid childhood growth were more consistently associated with earlier pubertal timing than were measures of psychosocial stress. We discuss future research directions to investigate the impact of psychosocial stress on pubertal timing more robustly, including methodological and mechanistic considerations, and contextualization of findings by socioecological environments.

## Graphical abstract

**Figure:**
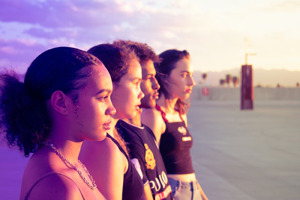

**Social media summary:** New paper ‘Psychosocial and energetic factors on human female pubertal timing: a systematized review’ by @GlassDelaney, Joy Geerkens and Melanie Martin. With systematic review results, they reflect on bio mechanisms and socioecological mediation of pubertal timing.

## Introduction

In human females, pubertal timing (referring to the onset and pacing of pubertal development) is proposed to be flexibly attuned to energetic resources and social cues in the environment (Ellison et al., 2012). Across populations, median ages at menarche range from around 7 to 16 (Ellison et al., 2012; Parent et al., 2003; Thomas et al., 2001). Within this range of variation, optimal pubertal timing represents a life history trade-off between energetic investment in continued growth vs. reproduction, with consequences for lifetime reproductive success (Charnov & Berrigan, 1993; Chisholm, Quinlivan, et al., 2005; Kramer & Ellison, 2010; Reiches et al., 2009; Stearns, 1992). Life history trade-offs are particularly pronounced in females, as higher oestrogen levels promote ovarian maturation and increased body fat promotes earlier closure of epiphyseal plates, leading to reduced growth in length/height (Rogol et al., 2002). Another trade-off is that earlier puberty can lead to earlier first reproduction and higher fertility, but at increased risk of maternal and offspring morbidity and mortality (Weibel et al., 2020). Conversely, continued body growth that delays reproductive maturity may increase maternal and offspring fitness, but decrease maternal lifetime fertility (Fraser et al., 1995; Stearns, 1992; Weibel et al., 2020; G. C. Williams, 1966).

Flexible pubertal timing within humans has been proposed to relate to energetic availability or sufficiency in childhood, wherein greater energetic resources favour earlier pubertal timing, and chronically low resources favour delayed or slower pubertal progression (Ellison, 1990, 1996; Wasser & Barash, 1983). However, research has also posited that greater childhood stress can accelerate pubertal timing, which may be evidenced by assessing differences in demographic patterning. Across species, demographic strategies exist along a continuum, occurring either relatively faster or slower (Charnov & Berrigan, 1993; Stearns, 1992). Faster strategies are described as rapid juvenile growth, earlier puberty, and smaller adult size, whereas slower strategies are characterized by slower juvenile growth, later puberty, and larger adult size (Charnov & Berrigan, 1993; Stearns, 1992). Humans and other animals who have long lifespans are particularly sensitive to harsh conditions earlier in life, which may shorten life expectancies (Douhard et al., 2014; Stearns & Rodrigues, 2020). Recent formal mathematical models contest earlier verbal models suggesting that extrinsic adult mortality selects for shorter lifespans and earlier senescence (Moorad et al., 2019). However, early life adversity has been shown to reduce life expectancies in humans and other primates (Weibel et al., 2020). Accelerated reproduction may be favoured when lifespans are shortened, highly dependent on local conditions (Nettle, 2011; Stearns, 1992). Thus, it was suggested that greater psychosocial stress experienced in early life (i.e. perceived threats or insults to survival or wellbeing) may favour a relatively faster life history strategy (Bateson et al., 2014; Belsky, 1991; Chisholm, Burbank, et al., 2005; Chisholm, Quinlivan, et al., 2005; Draper & Harpending, 1982; Ellis, 2004). The Psychosocial Acceleration Theory (Acceleration Theory hereafter) proposed by Draper and Harpending (1982) and expanded on by Belsky (1991) suggests that early life adversity cues developmentally plastic responses. For example, many researchers have posited that the stress (Ellis & Garber, 2000; Moffitt et al., 1992) or resource limitation resultant from father absence may promote earlier puberty (Belsky, 1991).

However, the relative effects of childhood psychosocial stress on pubertal timing may be confounded. First, confounding may occur when poverty is associated with both rapid growth or obesity and high psychosocial stress. For example, in high resource environments such as the USA, disadvantaged socioeconomic status (SES) is generally associated with greater risk for overweight/obesity and more rapid physical and sexual maturation (Bleil et al., 2017; Braithwaite et al., 2009; Deardorff et al., 2011; Obeidallah et al., 2000). Socioeconomic status is a major driver of health inequalities, and obesity rates are higher among disadvantaged SES groups because of nutritional excess, processed foods, and food insecurity, as well as a more sedentary lifestyles (owing to a lack of safe places for physical activity; Fleming et al., 2021; Working Committee on Social Determinants of Health and WHO, 2008; Oberle et al., 2019; D. R. Williams et al., 2019). Culturally specific or place-based social stratification may play a role in differentiating psychosocial and energetic exposures one experiences in childhood. These have the potential to impact growth and pubertal timing and we suspect that they probably exert effects in concert with each other. For example in the USA, racism has structured discriminatory access to resources, which impacts risks of living in poverty, disadvantaged SES and experiencing heightened psychosocial stress (Sant et al., 2021; Semega et al., 2017; Trent et al., 2019). Thus differences in pubertal timing among racialized and historically excluded groups probably reflect effects of structural racism (Bleil et al., 2017).

Conversely in other populations high psychosocial stress and energetic scarcity may co-occur. For example, rates of obesity have typically been higher among *more* advantaged SES groups in lower and middle-income countries (McLaren, 2007; Sear et al., 2019; Wang, 2001; A. S. Williams et al., 2018). Within these countries, social inequalities have been associated with growth stunting or poor growth outcomes among more disadvantaged groups, rather than rapid growth (B. A. Bogin & MacVean, 1978; Candler et al., 2017; Monteiro et al., 2010). However, these patterns are shifting because obesity is increasingly prevalent among disadvantaged, middle and elite classes in low and middle income countries in both childhood and adulthood (Monteiro et al., 2004).

Beyond population differences in rates of obesity and social inequalities, environmental mismatch may help explain why the effects of childhood growth and psychosocial stress on pubertal timing are so difficult to differentiate. In ancestral environments, psychosocial stress was probably paired with energetic exertion (Lee et al., 2016), whereas psychosocial stressors in some high resource settings coexist with high-fat, high-carb diets (Stearns & Rodrigues, 2020). Thirdly, there may be residual confounding wherein psychosocial stress is directly impacting child growth and therefore pubertal timing. Thus, while ample research has demonstrated associations between energetic factors and psychosocial stressors on pubertal development, it is unclear the extent to which they have addressed potential confounding by assessing these factors simultaneously.

This systematic literature review seeks to determine if and to what extent existing research on pubertal timing has simultaneously examined the influence of energetic and psychosocial factors. First, we summarize the energetic and psychosocial frameworks applied to human female pubertal development, including the mechanisms proposed by which energetic availability and psychosocial stress may promote more rapid pubertal development, evidence for these associations, and critiques of observational research in these areas. We then present results from an unregistered systematic review conducted with the following aims: (a) to identify original studies that have assessed both energetic and psychosocial influences on pubertal timing; and (b) where possible, to compare the relative effects of energetic vs. psychosocial influences on earlier pubertal development.

## Background

### Biology of puberty

Puberty is the short-term shift (over months to years) from a childhood state of negative feedback in gonadal hormones on the hypothalamic–pituitary–gonadal (HPG) axis to positive feedback loops; adolescence is the more than 6 year transition from a pre-reproductive to an adult reproductive status (B. Bogin, 2015). Throughout puberty, body composition, stature and secondary sexual characteristics dynamically transform. This process begins when gonadotropin-releasing hormone resumes a pulsatile pattern after being dormant in childhood (Reiches, 2019). The HPG axis is responsible for ‘switching on’ gonadotropin-releasing hormone and it regulates the production and release of gonadal hormones such as oestrogen and testosterone, in addition to growth hormone (Reiches & Ellison, 2022). These hormones help coordinate increases in linear growth, peripheral fat, lean muscle mass, body hair and reproductive organ development, as well as menarche (Ellis, 2004; Nepomnaschy et al., 2020; Reiches, 2019). Alongside these changes, the hypothalamic–pituitary–adrenal axis (HPA) is subtly changing because areas of the brain related to HPA responsivity and reactivity (e.g. the amygdala, prefrontal cortex, and hippocampus) substantially mature during puberty (Romeo, 2010). This results in potentially heightened biological stress responses governed by the HPA (Romeo, 2010). The pubertal transition requires allocation of environmental and somatic resources (e.g. food, body fat) to increase growth and reproductive development, coordinated by a complex interplay of energy balance and multiple physiological, metabolic and neuroendocrine systems (Ellis, 2004; Ellison, 2017; Reiches, 2019; Reiches & Ellison, 2022).

### Energetics hypotheses

Evolutionary theorists suggest that pubertal timing is facultative at the species level (MacDonald, 1999; Stearns, 1992; Surbey, 1990; Wasser & Barash, 1983). There is robust evidence that pubertal onset (from relatively early to more delayed on a continuum) is highly heritable, with heritability commonly estimated greater than 0.40 (Eaves et al., 2004; Howard, 2019; Morris et al., 2011; Zhu et al., 2018). However, because a single genotype can exhibit myriad phenotypic expressions (‘norm of reaction’), the probabilistic impact of genetics on pubertal timing may well depend on local socioecological conditions (Ellis, 2004; Mcintyre & Kacerosky, 2011; Stearns & Koella, 1986; Worthman et al., 2019). For example, a large norm of reaction has been observed for age of first menstruation, with median ages at menarche ranging from 12.5 in post-industrial contexts to 18+ in other horticultural contexts (Mcintyre & Kacerosky, 2011; Worthman et al., 2019). Thus, local energetic conditions may influence where an individual falls within the reaction norm.

**Figure 1.**
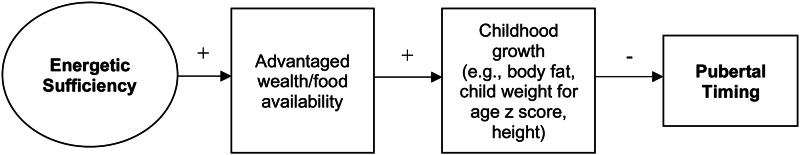
Energetic model of pubertal timing

*Energetics theory of timing of pubertal development* (henceforth Energetics Theory) proposed that natural selection favoured mechanisms coordinating pubertal timing to signals of resource sufficiency or availability (Ellison 2001, Ellis 2004). The pubertal trade-off (i.e. invest in reproductive development now or wait) is influenced by the presence of a metabolic surplus wherein metabolic energy exceeds the costs of maintenance (Ellison, 1990, 2001). As depicted in Figure 1, energetic or resource sufficiency is the latent variable driving Energetics Theory. What is observed to capture energetic sufficiency is typically wealth, food or nutritional availability or adequacy. Moreover, advantaged wealth or food availability/adequacy is predicted to positively impact (+) childhood growth, which will result in earlier pubertal timing (−) (see Figure 1).

Ideally, to measure energetic status or energetic sufficiency, researchers should utilize multiple longitudinal measures of childhood growth that can capture trajectories of both prior and current conditions. Measures of longitudinal growth or size that can be observed are changes in height and weight. Variability in height in puberty is overwhelmingly attributed to variation in growth tempo (which characterizes how fast or slow someone is growing relative to others; Hermanussen, 2016). Height is also useful to characterize the pubertal linear growth spurt, such as measuring peak height velocity, incremental changes in height over a given period or growth tempo. It is therefore critical to have repeated measurements when assessing pre-pubertal and pubertal growth because prior conditions will impact current conditions, as an individual's developmental pace tends to stay consistent across child and adolescent development (Hermanussen, 2016). However, there are some limitations of using anthropometric indicators such as height to proxy resource sufficiency. While instances such as starvation do result in linear growth deficiencies, recent analyses of historical data suggest that relationships between height and nutrition may not be as causally linked as once considered (Hermanussen & Wit, 2017). Moreover, height may be highly heritable whereas weight or body mass index (BMI) may be less heritable, which suggests that weight or BMI may be more sensitive to current or changing energetic conditions (Wainschtein et al., 2021; Yang et al., 2015; Yengo et al., 2018).

Both static measures of body weight and BMI may prove useful, at least at the population level, to proxy current energetic conditions. Weight and change in weight over time can signify nutritional status (Cole et al., 2005). Body mass index strongly correlates with total body fat percentage and it is a reasonable proxy for accrued resource availability in young females (Bygdell et al., 2021; Dietz & Bellizzi, 1999; Goulding et al., 1996; Hall & Cole, 2006; Widhalm et al., 2001), especially when it is used longitudinally to understand composite measures of body size changes over time (Nuttall, 2015). However, associations between exact body composition (i.e. fat mass) and BMI are weak and unreliable at the tails of the BMI distribution or outside of the ‘normal’ BMI range when using it as a categorical variable (Goulding et al., 1996; Woo, 2009). With this in mind, it is better in the context of pubertal timing and energetic stores to use BMI as a continuous variable (raw units), not binned into categories (i.e. ‘normal’ or ‘underweight’). In doing so, relative developmental pace and prior conditions can be tracked, whereas a BMI *Z*-score for age may be a more suitable measure in contexts where repeated measures are not attainable (Cole et al., 2005). Researchers are often constrained to limited sampling frequency and therefore may rely on comparing categorical BMI or height/weight for age *Z*-scores. While these may be appropriate for capturing current energetic conditions, longitudinal sampling necessarily allows observation of energetic status fluctuations, proxied by childhood growth.

Epidemiological and historical evidence further suggest that rapid childhood growth as well as adequate or over-nutrition are associated with earlier pubertal timing (Ahmed et al., 2009; Biro et al., 2006; Cho et al., 2010; Durda-Masny et al., 2019; Hossain et al., 2010; Ossa et al., 2010; Parent et al., 2003; Pasquet et al., 1999). For example, in a large Swedish population-based study, higher BMI (used as a proxy for nutritional status) measured at ages 2–8 was associated with greater increases in height attainment in the same period and earlier pubertal timing, but it was not associated with final height (He & Karlberg, 2001). A recent analysis of cohort trends from 27 low- and middle-income countries demonstrated that age at menarche has broadly declined, probably resulting from a combination of changes in nutrition, reductions in physical activity and changes in wealth distributions (Leone & Brown, 2020). However, the rate of decline varied by location; for example, in Egypt and Tunisia, menarcheal trends were flatter (e.g. stalling), whereas in the Philippines and Colombia rapid declines were present. Leone and Brown (2020) similarly found that poorer individuals had earlier menarche in earlier surveys, whereas in later surveys richer individuals had earlier menarche in Yemen and the Philippines, but a reverse trend was found in Egypt. Furthermore, evidence from Brazil, India and Pakistan also suggests that undernutrition or malnourishment (when in comparison with adequately nourished individuals) may delay pubertal timing (Barros et al., 2019; Campisi et al., 2021; Qamra et al., 1990; Satyanarayana & Naidu, 1979).

Similar associations have been detected longitudinally between food insecurity and later ages at menarche in Colombia with over 15,000 participants (Jansen et al., 2015a) and in Ethiopia (Belachew et al., 2011) with 900 participants. However, in the USA, food insecurity or inadequate nutrition has been associated with earlier puberty (M. Burris et al., 2020; M. E. Burris & Wiley, 2021). This counterintuitive observation may suggest that pubertal timing is delayed by more extreme or sustained nutritional deprivation than that experienced by marginalized US children who may face nutritional insecurities of excess processed foods (Ellis, 2004; Kirkwood et al., 1987). While there are certainly heterogenous populations in the USA and other Western countries, very little research (to our knowledge) has been conducted on pubertal development in subpopulations within these contexts where more sustained nutritional and psychosocial deprivation may be occurring, for example among unsheltered teens or children in immigration or punitive detention centres.

### Psychosocial stress hypotheses

**Figure 2.**
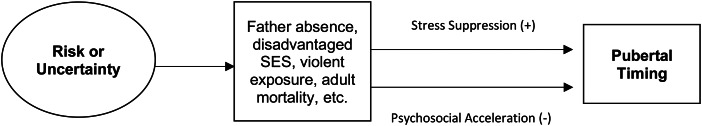
Psychosocial model of pubertal timing.

Energetics Theory was extended to include psychosocial stressors in Stress Suppression Theory (henceforth Suppression Theory; Ellis, 2004), i.e. chronically low energetic availability *or* psychosocial stress insults would act as a cue to delay pubertal timing until conditions improved (Cameron, 1997; MacDonald, 1999; Miller, 1994; see Figure 2). Mechanistically it was proposed the HPG is *suppressed* via co-activation of corticotropin-releasing hormone and locus coeruleus–norepinephrine, the HPA, and the autonomic nervous system until there are improvements from psychosocial stress insults *or* greater energetic availability (Ellis, 2004; McEwen & Seeman, 1999; Rivier et al., 1986). Evidence for the HPG being suppressed in response to stress has come from observations of HPG suppression in chronically stressed adult females (Ellison, 2001; Iwasa et al., 2018; Nappi & Facchinetti, 2003), although it is less clear if HPG suppression occurs in adolescence in response to diverse forms of psychosocial stress (Shirtcliff et al., 2015).

Many studies testing Suppression Theory in non-human animals have examined correlations between stressful situations such as antagonistic encounters and HPA activation and HPG suppression (Ellis, 2004; Rivier et al., 1986; Viau & Sawchenko, 2002). In primates living in dominance hierarchies, it was proposed that being lower ranked would be stressful and result in higher circulating glucocorticoids as well as suppression of pubertal development (Blanchard et al., 2001; Cameron, 1997). However, Creel (2001) found that basal and circulating glucocorticoids were too variable across species and across differently ranked individuals to warrant evidence for this hypothesis. Importantly, Creel (2001) explained that social dominance hierarchies evolved to mitigate highly costly conflicts and intergroup escalations, and there would be no expectation of higher glucocorticoid levels in less dominant primates (Creel, 2001). There is currently no consensus about whether being lower or higher ranked in social hierarchies is inherently more stressful (and reflected in glucocorticoid variability) across species (Beehner & Bergman, 2017; R. M. Sapolsky, 2005).

**Figure 3.**
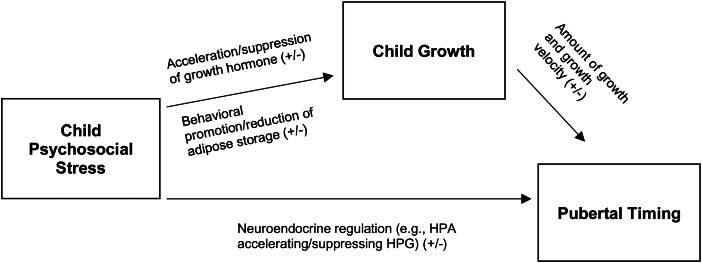
Proposed biological mechanisms between psychosocial stress, growth and pubertal timing.

However among humans who do not exist in strict dominance hierarchies but rather in a web of hierarchies, SES has been robustly associated with psychosocial stress profiles and disease outcomes (R. Sapolsky, 2005; R. M. Sapolsky, 2005). For example, in a systematic review of 36 studies, Niere et al. (2020) suggests that psychosocial factors (emotional and social) may influence linear growth in childhood depending on the environment. Among the studies they reviewed, they report that advantaged socioeconomic status, social positioning, and parental education are associated with more rapid growth tempo whereas disadvantaged SES, parental education and social mobility were associated with slower growth (Niere et al., 2020). Citing evidence from studies of emotional deprivation (Spencer, 2017), social isolation of migrant children (Özer & Scheffler, 2018) or childhood institutionalization (Kroupina et al., 2015), Niere et al. (2020) and B. Bogin (2021b) suggest these types of psychosocial stressors may also suppress growth hormone (via a biological stress response) resulting in poorer growth outcomes (B. Bogin, 2021b; see Figure 3). However, as they note, stress responses are modulated by stability of the environment, which is notoriously difficult to capture in humans (Creel et al., 2013; Knight & Mehta, 2017; Niere et al., 2020; R. M. Sapolsky, 1992).

One striking context where Suppression Theory may be invoked to explain variable pubertal timing is among individuals experiencing eating disorders such as anorexia nervosa or avoidant/restrictive intake disorder. These are characterized by very low or infrequent energy intake or caloric restriction, weight suppression (Lowe et al., 2018) and the co-occurrence of high psychosocial stress or psychological comorbidities like depression and anxiety (Becker et al., 2019). In this situation, chronically low energy intake paired with high psychosocial stress has been shown to delay puberty, with the caveat that catch-up growth may occur with potentially negligible impacts on final adult height (Neale et al., 2020).

Overall, however, it appears that psychosocial stress has rarely been shown to suppress pubertal development in human studies, despite observations of suppression in adulthood (Shirtcliff et al., 2015). The extent to which psychosocial stress impacts pubertal timing may also depend on the timing, duration and biobehavioural needs of the individual (Shirtcliff et al., 2015). For example, it has been proposed that psychosocial stressors may exert smaller effects later in puberty compared with pre-puberty, because (a) stress exposures pre-puberty may matter more for developmental plasticity of puberty and (b) by the time puberty is well under way, energetic and neuroendocrine reorganization (e.g. increases in gonadal and steroid hormones, physiological changes) may limit the effects of stressors (Phan et al., 2021; Shirtcliff et al., 2015). Thus, puberty may be a particularly unique period wherein stress co-occurs with, rather than inhibits, gonadal hormone production, via synthesis of the HPG–HPA axes; this ‘hormonal coupling’ can be tested by tracking changes in the trajectories gonadal and steroid hormones across puberty (Marceau et al., 2014, 2015; Shirtcliff et al., 2015).

In contrast to Suppression Theory, Psychosocial Acceleration Theory (Acceleration Theory furthermore) posits that stressful early life ecological and familial conditions favour accelerated pubertal development (Draper & Harpending, 1982). We highlight in Figure 2 that risk or uncertainty in the environment is the latent variable driving Psychosocial Acceleration Theory and Stress Suppression Theory. Examples of observed psychosocial stressors indicative of risk or uncertainty may include father absence, disadvantaged SES, and exposures to violence. How observed psychosocial stress impacts pubertal timing, either earlier (−) or later (+) depends on the socioecological context (see Figure 2). Belsky (1991) originally hypothesized that internalizing behaviour in response to perceived stress (e.g. social withdrawal, nervousness, irritability, eating more/less) in females promoted fat storage and lowered metabolism, resulting in more rapid growth, but did not elucidate mechanisms for this (Belsky, 1991). It is, however, plausible that heightened psychosocial stress may result in disordered eating patterns, reduce physical activity and result in more rapid weight gain and growth. Biobehavioural factors such as appetite (operating through ghrelin or leptin) and sociocultural diet expectations (Mousa et al., 2010) may also have an impact on energy intake or adiposity, with downstream consequences for pubertal timing (Michels, 2019). Chisholm, Burbank et al. (2005), Chisholm, Quinlivan, et al. (2005) and Belsky et al. (2015) later suggested that chronic activation or greater elevation of basal cortisol levels may be one mechanism through which stress accelerates sexual development, invoking the Adaptive Calibration Model (Belsky et al., 2015; Chisholm, Burbank, et al., 2005; Chisholm, Quinlivan, et al., 2005; Del Giudice et al., 2011; Doom & Gunnar, 2013). Chisholm, Burbank et al. (2005) and Chisholm, Quinlivan, et al. (2005) further emphasized that because individuals are acclimated to different sociological environments, pubertal timing will exhibit intra- and inter-individual variation in relation to stress reactivity.

Acceleration Theory has frequently examined parental investment as a primary cue of socioecological conditions, proposing that low parental investment during childhood is perceived as a reliable cue of low future investment (Belsky et al., 1991). In such circumstances, earlier reproduction is favoured as delayed reproduction would not benefit from future accrued parental resources (Belsky, 1991; Ellis, 2004). Many studies testing Acceleration Theory have detected associations between early life adversity (e.g. family conflict, father absence, parental attributes) and earlier pubertal timing (Belsky et al., 2007, 2015; Ellis et al., 2011; Moffitt et al., 1992; Tremblay & Frigon, 2005). Associations between father absence and earlier pubertal timing have been robust, and it has been suggested that this may result from a higher likelihood of unstable relationships and parenting behaviour in adulthood (Belsky, 1991; Draper & Harpending, 1982). However, Sear et al. (2019) cautions that the duration of father absence, the reason for his absence (e.g. death, divorce, never having been present) and the absence of other family members may generate varied effects on pubertal timing. Furthermore, in environments characterized by low mortality, high energetic availability, normative conventions of a nuclear family and established secular trends of earlier development, associations between father absence and earlier puberty are robust (Sear et al., 2019). In all other contexts where there is not a normative nuclear family structure, higher mortality and lower energetic availability, father absence has been an inconsistent predictor of pubertal timing if at all (Sear et al., 2019).

Mixed cross-cultural evidence for a relationship between father absence and earlier pubertal timing may suggest that psychosocial stress distinctly correlates with resource availability across varied socioecological contexts. For example, father absence in contexts of normative nuclear family structures may threaten resource availability or social network connections, leading to psychosocial stress, whereas in other contexts, the father's impact on child wellbeing may be minimal or easily substituted (Sear et al., 2019). It is also possible that across contexts, psychosocial and resource environment factors interact in complex ways. For example, Hulanicka (1999; Hulanicka et al., 2001) found that among disadvantaged SES Polish female adolescents, poverty (resource scarcity) delayed menarcheal timing overall. However among disadvantaged SES adolescents, greater chronic psychosocial stressors (parental death and divorce, familial deviance like alcoholism, prolonged illness of family member) were associated with earlier menarche (Hulanicka, 1999; Hulanicka et al., 2001). The latter implies that the effect of psychosocial stress may outweigh the effect of resource scarcity in this context, which would otherwise delay menarcheal timing.

With Energetics Theory, Suppression Theory and Acceleration Theory in mind, there are conceptual, theoretical and methodological critiques that have emerged in recent years that warrant further discussion. First, one of the tenets underpinning Acceleration Theory is the idea that a fast–slow life history continuum can be applied to individuals. However, statistical associations between measures of greater psychosocial stress and earlier pubertal timing are not necessarily evidence of an evolutionary strategy favouring a fast life history (Sear, 2020; Stearns & Rodrigues, 2020). Variable reproductive strategies may simply represent normal variation within a reaction norm (Stearns & Rodrigues, 2020). Moreover, Stearns and Rodrigues (2020) argue that the fast–slow continuum applies when comparing demographic life history patterns across species, but not among individuals. In experiments citing evidence of selection for faster reproduction within a population, adult mortality rates, not juvenile cues of a risky environment, were key selective factors (Stearns & Rodrigues, 2020).

Furthermore, pubertal timing is assumed to be associated with timing of other reproductive outcomes such as first intercourse and pregnancy that may influence lifetime reproductive success, yet few studies have actually tested this assumption with sufficient biodemographic data, let alone in relation to psychosocial stressors (Sear, 2020). Leveraging over 48 years of continuous longitudinal data among wild Amboseli baboons in Kenya, Weibel et al. (2020) tested Nettle and Bateson's Internal Predictive Adaptative Response model (Nettle & Bateson, 2015) to probe the adaptive benefits of accelerated reproduction in light of experiencing early life adversity (Weibel et al., 2020). They found that early life adversity predicted shorter lifespans, but it did not accelerate reproduction. Furthermore, accelerated reproduction was positively associated with greater lifetime reproductive success only in baboons that lived longer lives (Weibel et al., 2020). In humans, it is still unclear whether earlier pubertal timing indeed reflects an adaptive strategy, and whether such an evolutionary strategy can be witnessed on an individual scale and how cues from the environment are meaningfully adopted to adjust bodily strategies. Amir et al. (2016) found that high subjective environmental risk and low access to economic resources predicted earlier menarche in US females similar to prior work (Šaffa et al., 2019). Their findings suggest that perception of local environmental risk or local life expectancies may provide further evidence of an individual strategy, however, to date the mechanisms are unclear.

Furthermore, psychosocial ‘stress’ is often broadly and differently defined across studies (Shirtcliff et al., 2015). Environmental cues that can be signalled via psychosocial stress and that are proposed to influence life history strategies include adult extrinsic mortality, resource availability, and predictability, which may be at odds with the vast types and definitions of psychosocial stress utilized in studies (Shirtcliff et al., 2015). This is compounded by the fact that few studies have tested Acceleration Theory or Suppression Theory while also accounting for biomarkers of stress or HPA activation/suppression (Negriff et al., 2015; Peckins et al., 2015; Saxbe et al., 2015). Some of these semantic, methodological tensions are also evident when using SES, which has been used as both a proxy for resource availability (specifically risk of food insecurity) and psychosocial stress. While we do not argue that SES is definitively a psychosocial stress variable, we believe that it may represent both the psychosocial and energetic conditions that humans are embedded in. Psychosocial stress developing from exposure to poverty or disadvantaged social status may not have a clear-cut suppressive effect on pubertal timing. Socioeconomic status may be confounded in environments where poverty is associated with both greater psychosocial stress and food insecurity, which may promote obesity (M. Burris et al., 2020; M. E. Burris & Wiley, 2021; Gundersen et al., 2011; R. Sapolsky, 2005; R. M. Sapolsky, 2005).

In other contexts, poverty *may not* be associated with obesity but there may be substantial differences in health and nutritional status by wealth or class such that individuals who belong to advantaged class groups or have greater wealth tend to mature earlier than individuals belonging to disadvantaged class groups (Jansen et al., 2015b; Karim et al., 2021; Montero et al., 1999; Padez, 2003; Rimpelä & Rimpelä, 1993; Shaik et al., 2016). A meta-analysis by Zhang et al. (2019) sought to quantify the effect of early life adversity (measured via total adverse childhood events) on pubertal timing. While total adverse childhood events were not associated with earlier pubertal timing across the 43 studies they reviewed, individual events such as sexual abuse, father absence and family dysfunction were associated with earlier pubertal timing and exhibited high heterogeneity. Thus, owing to heterogeneity among existing evidence along with the rationale discussed above, there are multiple potent reasons why energetic status should be accounted for in studies of psychosocial stress and pubertal timing (Zhang et al., 2019).

## Methods

### Systematized review aims and protocol

The primary aim of this review was to identify studies that simultaneously assessed the effect of psychosocial stress and childhood energetic status on pubertal timing, and critically evaluate the relative strength of the evidence for these respective factors across selected studies. We designed an unregistered systematic review protocol based on select PRISMA guidelines (what we term a *systematized* review). Following this protocol, we: (a) specified aims for the review; (b) identified data sources; (c) determined exclusion and inclusion criteria; and (d) validated concept maps and search strings before conducting the search. We did not attempt to discern risk of bias – typically operationalized as a qualitative or quantitative assessment of study design quality (Moher et al., 2009) – due to the broad inclusion criteria we defined. A metanalysis was not possible owing to methodological differences across studies, including how factors were operationalized, analysed and reported. The search was conducted in April 2019 across PubMed, Web of Science, JSTOR, ProQuest and APA PsycInfo databases.

### Search strategy

We created concept maps to produce a list of search strings for each individual database. These were tailored according to the specific database search style. We identified Ellis and Essex (2007) as a ‘key’ study to validate our search strings for returning relevant studies. We also used Jean et al. (2011) and Amir et al. (2016) to ensure that our search strings were returning relevant results in theme or scope, given that these were known studies on pubertal timing (Amir et al., 2016; Jean et al., 2011). A complete list of search strings for each database and the full protocol for this systematized review are available on Open Science Framework (https://osf.io/nckrh/). For every search string developed, we used search terms or check boxes to specify prospective or retrospective pubertal staging and psychosocial stress measures but only prospective childhood energetic measures. For example, the search string used for PubMed was (‘biosocial’ OR ‘Socioeconomic Factors’[MeSH Terms] OR ‘psychosocial’ OR ‘early life adversity’ OR ‘energetic stress’ OR ‘father absence’ OR ‘sexual abuse’) AND (‘Body Composition’[MAJR] OR ‘growth’ OR ‘height’) AND (‘Menarche’[MAJR] OR ‘Menarche/physiology’[MAJR] OR ‘pubertal timing’ OR ‘reproductive timing’) AND (longitud* OR prospective OR cohort OR ‘Multivariate Analysis’[MeSH Terms]) AND (‘Adolescent’[MeSH Terms]).

JG conducted the initial title search using final search strings across five databases. A total of 1868 titles were returned, with 1069 remaining after removing duplicates: PubMed, *n =* 236; Web of Science, *n =* 85; JSTOR, *n =* 392; ProQuest, *n =* 310; APA PsycInfo, *n =* 46.

### Screening

**Figure 4.**
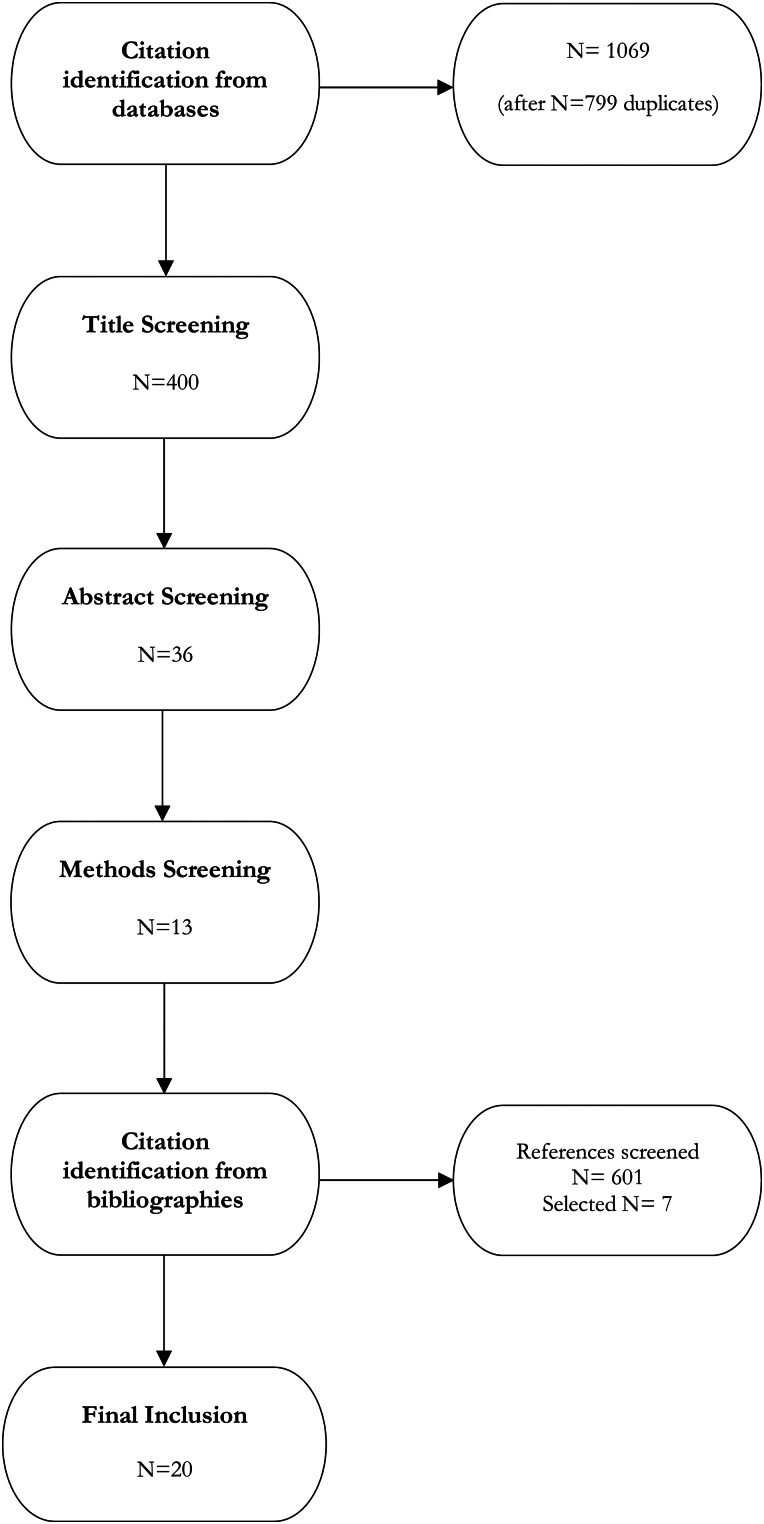
Flow chart of systematized review process.

From the 1069 non-duplicate titles returned, JG selected 400 titles for further review based on qualitative assessment of title relevance. If titles were vague, she quickly reviewed abstracts for more information. DG and JG independently reviewed abstracts and methods from the title selection. DG and JG selected 36 abstracts from the 400 selected titles based on inclusion and exclusion criteria (Table 1). If it was unclear whether the abstract met our criteria, the article was retained until further screening. After the abstract selection, JG and DG performed a methodology screening and selected 13 papers for review. Conflicts in study selection at all stages were resolved by MM. DG, JG, and MM then independently screened references of selected studies (*n =* 601), selecting seven additional papers for critical review after consensus discussion. In total we selected 20 papers for critical review (Figure 4), representing 1.25% of non-duplicate titles returned from the initial search.

**Table 1.** Inclusion and exclusion criteria

Inclusion criteria	Exclusion criteria
Written in English Human female participants Study design included measures of both energetic and psychosocial factors taken prior to pubertal outcome Energetic and psychosocial factors assessed from at least ages 5–7 years or older Original peer-reviewed research	Non-human animals Human males Assessed only energetic or psychosocial factors but not both Retrospective studies without energetic factors measured prior to pubertal outcome (e.g. menarche), or status quo studies without sufficient pre-outcome subjects Energetic or psychosocial factors assessed only through early childhood Systematic reviews or dissertations Studies that only assessed nutritional status (e.g. kcal/day) but not anthropometrics Studies that only assessed hormonal cut-offs for pubertal staging

The primary inclusion criteria for selected studies at all stages (Table 2) were assessment of both energetic and psychosocial factors in relation to pubertal outcomes. To avoid confounding owing to weight and adipose tissue gain after mid-puberty in females, we included only studies that measured energetic factors prior to the specified pubertal outcome, or that used the status quo method with relatively sufficient numbers of individuals falling into pre- and post-outcome status categories. Acceptable energetic measures included anthropometrics such as body fat percentage, height, weight, height or weight increase, height or weight velocity, skinfold measurements or body mass index. We accepted pubertal outcomes observed prospectively, by status quo methods, or retrospectively if energetic factors had been measured prior to outcomes. Acceptable pubertal measures included age at menarche, menarcheal status, Tanner Stages, height velocity or other parameters of pubertal timing or relative pace of development. We accepted psychosocial factors measured prospectively, by status quo or retrospectively (e.g. father absence at age 5, reported at time of pubertal outcomes). Psychosocial criteria were intentionally kept broad but specific measures such as early life adversity, socioeconomic status, psychosocial (broad MESH term), father absence and sexual abuse were included in search strings. In general, any studies with factors conceived of as psychosocial variables according to the authors were acceptable for inclusion.

**Table 2.** Summary of author aims, location, study size, design and measurement of independent and dependent variables among the 20 selected studies

Study reference	Author aims	Location	Study size	Study design	Pubertal outcome	Anthropometric measures	Psychosocial measures
Adair (2001)	Examine intrauterine growth and age at menarche; authors emphasize prenatal environment in disease risks	Philippines	*n =* 966	Self-report at visit, survey at age 8	Age at menarche (AM)	Height, weight, skinfold at 8 years, caloric intake	Household wealth, ownership and quality, maternal education
Akther and Islam (2012)	Examine secular decline in menarche and sociodemographic risk factors in non-Western context	Bangladesh	*n =* 248	Status-quo surveys ages 10–14	AM	Height, weight, BMI	Parental income and education, number of siblings, birth order
Blell et al. (2008)	Simultaneously examine effects of early life factors on age at menarche. Authors focus on risks of breast cancer and low bone mass	England	*n =* 882	Self-report ages 49–51, school health visit age 9, psychosocial birth until 5–9	AM	Height and weight	SES (parental occupation), adverse life events, household conditions
Braithwaite et al. (2009)	Investigate timing of menarche in Black and white females in relation to socioeconomic variables	USA	*n =* 2078	Annual self-report from ages 9–10 until ages 19–20	AM	Skinfolds, height, weight, upper thigh circumference, physical activity, caloric intake	Household income, parent education, family discord scale
Deardorff et al. (2011)	Examine father absence on sexual maturity. Authors utilize LHT and predict HPG activation is linked to fat deposition and BMI will mediate effects of father absence on thelarche	USA	*n =* 444	Annual clinic visit ages 6–8, follow-up 2 years later, caregiver report at visit	Tanner Stage (TS) (breast and pubic)	Height and weight	Father absence, annual income, birth order, housing conditions
Ellis and Essex (2007)	Authors test Acceleration Theory, hypothesizing the quality of paternal investment uniquely predicts timing of puberty separate from maternal investment. They predict BMI will partially mediate the effects of stressors on pubertal development	USA	*n =* 180	Parental/self-report at age 11, DHEA at age 7, anthropometrics ages 7–10, preschool survey	Salivary DHEA (Dehydro epiandrosterone); Tanner Stages (breast and pubic), Pubertal Development Scale	Height and weight	Family environment (10 measures of parent–child and marital relationships, psychopathology), SES
Graber et al. (1995)	Authors interested in bidirectional relationship between hormones and behaviour (authors term psychopathologies)	USA	*n =* 75	Status quo at annual clinic visit ages 10–14, annual questionnaire	AM, TS (breast)	Height and height for weight	Psychological adjustment (Youth Self Report scale), stressful life events (school, peer, family), father absence
Khan et al. (1996)	Examine linear growth in relation to age at menarche. Authors hypothesize linear growth stunting summarizes environmental, health and nutritional effects in childhood and is a strong predictor of menarche	Guatemala	*n =* 497	Height and household surveys ages 3–7 annually; AM by recall ages 15–30	AM	Skeletal growth (radiograph) until age 7, height 3–7 years	Household possessions and conditions
Kirchengast and Bauer (2007)	Assess independent effects of socioeconomic factors and body compositional factors on age at menarche	Austria	*n =* 1752	Status quo premenarcheal and anthropometrics, recall AM for post-menarcheal girls, ages 12–18	AM	BMI, lean body mass, absolute and relative fat mass	SES (via parental education andthe school type: grammar vs public school)
Kryzanowska et al. (2016)	Re-examine biosocial determinants of age at menarche using a different methodological approach than previous researchers Boynton-Jarrett and Harville (2012)	England, Wales, and Scotland	*n =* 4483	Recall age 16, anthropometrics age 7	AM	Height and weight	Free school meals, financial difficulties, social class (fathers’ profession, mobility, household crowding, birth order, number of siblings (authors term: family size)
Kyweluk et al. (2018)	Utilize LHT (Life History Theory) to investigate the effect of early life stressors on maturational timing in a population without co-occurring cues of environmental harshness or unpredictability, and nutritional abundance	Philippines	*n =* 807	Self-report at follow up ages 11.5–19.5, anthropometrics age 8.5	AM	Height, weight, caloric intake	Paternal instability (father absence up to 11.5years, mother unmarried in infancy); sibling death until 11.5 years, maternal absence (8.5 or 11.5 years)
Moffit et al. (1992)	Test the Psychosocial Acceleration Hypothesis proposed by Belsky, Steinberg and Draper	New Zealand	*n =* 416	Recall at age 15, anthropometrics ages 7, 9, 13 and 15. Psychosocial questionnaire age 7	AM	Weight	SES, father absence (status quo), family conflict and problem behaviours in childhood
Mulligan et al. (1999)	Determine the timing, magnitude and duration of the pubertal spurt for short normal and average height females, comparing to Tanner's standard. Authors critique the use of 1966 standard Tanner Stages considering secular changes in menarche	UK	*n =* 101	Status quo ages 8–14, anthropometrics every 6 months age 8+, parent interview ages12–14.15	AM, peak height velocity	BMI, height, weight	SES (based on the occupational status of the chief income earner in the household)
Nieczuja-Dwojacka et al. (2018)	Identify differences in body structure between early, average and late maturing females, taking into account family characteristics assessing socioeconomic and living conditions	Poland	*n =* 788	Status quo/self-report in questionnaire, anthropometrics each visit ages 11–19	AM	Height, weight; subcutaneous fat fold thickness, arm circumference	Parental education/profession
Orden et al. (2011)	Determine menarcheal age in urban schoolgirls and to identify associations with biological and socioeconomic variables; authors interested in temporal vs. spatial dimensions of menarche	Argentina	*n =* 1221	Status quo ages 9–15, psychosocial parental questionnaire	AM	Height, sitting height, weight, arm circumference, skinfolds	SES (parental occupation and education, family size, household characteristics)
Rao et al. (2011)	Understand height velocity and body fat impacts on age at menarche, accounting for socioeconomic status	India	*n =* 533	Status quo ages 9–16, anthropometrics 6 measurements ages 9–16, psychosocial questionnaire	AM	Height, weight, triceps skinfold thickness	High/low SES, family size/parent education
Reagan et al. (2012)	Utilizing a developmental perspective to understand health disparities, the authors seek to understand why menarcheal age differs between racialized (authors term racial) groups	USA	*n =* 2337	Maternal report aged 8–14, anthropometrics age 6, psychosocial recall ages 0–5	AM	BMI *Z-*scores at age 6	Poverty ages 0–5; % time living in poverty
Stark et al. (1989)	Estimate the incidence of early menarche. Authors use relative weight as an indicator of nutritional status. Written when evidence of nutritional status affecting sexual maturation was circumstantial	England, Scotland, Wales	*n =* 4427	Recall age 16, anthropometrics ages 7, 11, 17. Psychosocial survey at every follow-up	AM	Height and weight	SES, education, health, family
Tan and Camras (2015)	Examine precocious puberty in adopted Chinese participants, hypothesizing that pre-adoption developmental delay and changes in sociocultural context contribute to accelerated pubertal timing	USA and Canada	*n =* 298	Maternal report ages 7.3–11.1, anthropometrics ages 3.2–6.9	Pre-pubertal development scale, AM	Height and weight	Parenting style (permissive/authoritarian), child behavioural problems (internalizing/externalizing), income
Windham et al. (2008)	Examine how environmental cues (i.e. maternal smoking) could disrupt endocrine function	USA	*n =* 1,556	Recall ages 20–30, anthropometrics ages 4 and 7	AM	BMI, height, and weight at ages 4 and 7	SES (education and income), family composition, few, or no siblings

### Data synthesis

The following information was extracted from selected studies: author names, study date, study location, study design type, author aims and operationalized measures for pubertal outcomes, energetic, and psychosocial factors (Table 2). We then summarized associations between psychosocial and energetic variables and pubertal outcomes observed across studies (Table 3). We separately evaluated studies in which energetic and psychosocial effects on pubertal outcomes were evaluated in the same statistical models (*n =* 11). For these studies, we report statistically significant and non-statistically significant parameter estimates and the type of statistical analysis used (Table 4). Search and inclusion criteria were intentionally broad, which maximized the range of studies included, but limited our ability to directly compare and synthesize findings across studies. We included them in Table 4 if a large proportion of participants were still premenarcheal or sampled at age ranges before the mean or median age of menarche reported for the sample population.

**Table 3.** Factors measured and their association with pubertal timing

Study	Energetic factors	Psychosocial factors
Adair (2001; table 1, Table 3)	↑ (Higher BMI, greater skinfold thickness) ↓ (Low fat diet) × (Higher energy intake)	↑ (Advantaged SES)
Akther and Islam (2012; tables 20 and 21)	↑ (Greater height, weight, BMI)	× (Income, number of siblings & birth order)
Blell et al. (2008; tables 3 and 4)	↑ (Higher BMI, greater weight and greater weight velocity)	× (SES, order of birth, housing conditions & adverse life events)
Braithwaite et al. (2009; table 1)	↑ (Greater height, weight, percentage body fat, skinfolds, upper thigh circumference, BMI) × (Caloric intake, physical activity)	↑ (Low-income white, high-income Black) × (Family discord, parental education, family environment)
Deardorff et al. (2011; table 3)	↑ (Higher BMI)	↑ (Father absence, lower parental income)
Ellis & Essex (2007; figure 1)	↑ (Higher BMI)	↑ (Greater parental supportiveness, advantaged SES)
Graber et al. (1995; table 1)	↑ (Greater breast development, higher ponderal index and weight) × (Body fat percentage)	↓ (No family conflict, greater parental supportiveness, lower depressive affect, internalizing and externalizing behaviours)
Khan et al. (1996; table 4, model 3)	↑ (Greater height)	× (SES)
Kirchengast and Bauer, 2007; table 3 and 4 for ages 11 and 12)	↑ (greater BMI, lean body mass, percentage body fat, weight)	× (parental education)
Krzyzanowska et al. (2016; table 2)	↑ (Greater weight, greater height)	↓ (Number of siblings) × (Social class, mobility, crowding, birth order, free school meals (did/did not), greater financial difficulties)
Kyweluk et al. (2018; table 2, model 4)	↑ (Higher BMI, caloric intake for body weight)	× (Father and mother absence, sibling death)
Nieczuja-Dwojacka et al. (2018; table 1)	↑ (Higher BMI, greater weight, larger fat folds) × (physical activity)	↓ (Presence of any siblings) × (Maternal and paternal education, stress at home or school)
Moffit et al. (1992; figure 4)	↑ (Greater percentage body fat, greater weight)	↑ (Greater family conflict, behavioural problems; father absence)
Mulligan et al. (1999; table 1)	↑ (Greater height, weight)	× (SES)
Orden et al. (2011; table 3)	↑ (*Z*-Score height for age, sitting height ratio)	↑ (Higher maternal education)
Rao et al. (1998; figure 5)	↑ (Higher BMI)	↑ (Advantaged SES)
Reagan et al. (2012; table 2, model 1)	↑ (Higher BMI)	**↑** (Percentage poverty (always/never) among white participants) × (Percentage poverty (always/never) among African American participants)
Stark et al. (1989: 384–385)	↑ (Greater height, weight)	× (SES)
Tan and Camras (2015: 236)	**↑** (Greater weight)	**↑** (Behavioural problems)
Windham et al. (2008; table 3)	↑ (Greater height, BMI)	↑ (Greater maternal education, maternal income; few or no siblings) × (Family income)

Key: ↑ = earlier development, ↓ = later development, × = no statistically significant relationship.

**Table 4.** Multivariate results

Study	Outcome	Statistical analysis; parameter	Statistically significant determinants	Non-statistically significant determinants
Adair (2001: table 3, model 4)	Age at menarche (AM)	Survival analysis; HR	SES, 1.36***; BMI age 8, 1.14***; sum skinfolds, 1.03*	Diet score (energy intake), 1.03
Akther and Islam (2012: table 21)	Menarche achieved (yes/no)	Multiple logistic regression; odds ratio	Height (vs. 126–135 cm) 136–145 cm OR = 4.54 (*t*); ≥145 cm + OR = 9.14** Weight (vs. 21–30 kg) 31–40 kg OR = 3.95*; ≥40 kg OR = 32.78***	Fathers income (vs. <$10,000), $10,000–20,000 OR = 0.79 (*t*); $20,000+ OR = 1111.52 (*t*). Father's education above HSC (vs. HSC) OR = 1.19 (*t*)
Ellis and Essex (2007; figure 1)	Secondary sexual characteristics and Tanner Stage	Path analysis; standardized *β* weights	BMI, 0.30***; SES, −0.22**; parental supportiveness, −0.22**; marital conflict/depression, 0.18* Note: BMI hypothesized as mediator	—
Khan et al. (1996; table 4; model 3)	AM	Regression; unstandardized coef (*b*)	HAZ <−2 standard deviations at 3 years (childhood stunting): *b* = −0.16(±0.08)**	SES: 0.08 [±0.09] (*t*)
Krzyzanowska et al. (2016; table 2)	AM	Multivariate regression; odds ratio and unstandardized coef (*b*)	Height, −0.012***; weight, −0.045;*** number of siblings (family size), 0.074**	Free school meals (‘no’), 0.91; financial difficulties, 1.00; crowding (reference <1 person), 1–1.5 persons, 1.04; 1.5–2 persons, 1.02; 2.01+ persons, 0.99; birth order (reference 1), 2, 1.02, 3+, 0.99
Kyweluk et al. (2018; table 2, model 4)	AM	Multiple regression; unstandardized coef (*b*)	BMI, −0.14 (±0.03)***; caloric intake for body weight, −0.47 (± 0.09)***	Paternal absence, −0.04 (±0.14) (*t*); maternal absence, 0.11 ± 0.26 (*t*); sibling death, 0.00 ± 0.11 (*t*)
Moffit et al. (1992; figure 4)	AM	OLS regression; unstandardized coef (*b*)	Family conflict at age 7, −0.11***; body fat age 9, –0.26 Father absence at age 7, −0.10***; mediation of weight at age 9, father absence in childhood, 0.02**	Mediation of weight at age 9; family conflict at age 7, 0.08 (*t*); behavioural problems at age 7, 0.10 (*t*)
Orden et al. (2011; table 3)	AM	Logistic regression; exp(*b*)	HAZ, 2.19***; *Z*-score sitting-height-ratio, 1.75***; mother's education (tertiary), 1.82**	Mother's education (secondary), 1.30
Reagan et al. (2012; table 2, model 1)	AM	OLS regression; coef (*b*)	BMI *Z*, −1.31*; percentage poverty (white), −5.48*	Percentage poverty (African American), 1.81
Tan and Camras (2015: 236)	Breast development	Multiple logistic regression; odds ratio	Body weight OR, 1.11 (1.02–1.20)**; child behavioural problems, 1.04 (1.00–1.08)*	—
Windham et al. (2008; table 3)	AM	Regression; unstandardized coef (*b*)	Maternal education (reference ≤8 years) 9–11 years, 0.28 [0.03–0.53]*; BMI at age 7 (reference quartiles 2–3), quartile 1, 0.24 [0.002 0.48]; quartile 4, −0.27 [−0.51, −0.02]*; height at age 7, −0.03 [−0.05 to −0.02]*; number of siblings at age 7 (reference 5+), 1–2, −0.35 [−0.65 to −0.05]*	Maternal education (reference ≤8 years), ≥12 years, 0.22 [−0.07–0.52]; family income (reference <4,000/year), 4000–6999/year, −0.14 [−0.42–0.13]; 7000–9999/year, −0.16 [−0.46–0.15]; >10,000/year, 0.42 [−0.77, −0.07] Number of siblings at age 7 (reference 5+), 0, −0.45 [−0.97– 0.06]; 3–4, 0.01 [−0.26–0.28]

Symbols: (*t*)= trend (*p* < 0.10); *p < 0.05; **p < 0.01; ***p < 0.001. Coef or *b*, linear regression; OR, odds ratio; OLS, ordinary least squares regression; exp(*b*), exponentiated *β*.

Format: Variable – parameter estimate [confidence interval if available] or parameter estimate (standard error estimate).

Abbreviations: HAZ, height-for-age *Z*-score; BMI, body mass index; SES, socioeconomic status.

## Results

### Search results and study characteristics

We identified 20 studies (<2% of non-duplicate search results returned) that simultaneously assessed the effects of psychosocial and childhood energetic status on pubertal timing (Table 2). Seven of these studies were designed primarily to investigate energetic effects on pubertal timing (Adair, 2001; Khan et al., 1996; Krzyzanowska et al., 2016; Mulligan et al., 1999; Nieczuja-Dwojacka et al., 2018; Rao et al., 1998; Stark et al., 1989), but analyses adjusted for or separately analysed psychosocial influences as well. Conversely, seven studies explicitly assessed psychosocial effects but adjusted for or separately analysed energetic factors (Braithwaite et al., 2009; Ellis & Essex, 2007; Graber et al., 1995; Moffitt et al., 1992; Reagan et al., 2012; Tan & Camras, 2015; Windham et al., 2008). Six of the reviewed studies addressed both sets of factors as primary study aims (Akther & Islam, 2012; Blell et al., 2008; Deardorff et al., 2011; Kirchengast & Bauer, 2007; Kyweluk et al., 2018; Orden et al., 2011). Five of the selected studies were motivated by or explicitly tested hypotheses from a Life History perspective such as Acceleration Theory (Deardorff et al., 2011; Ellis & Essex, 2007; Graber et al., 1995; Kyweluk et al., 2018; Moffitt et al., 1992). The other 15 papers utilized epidemiological approaches to understand relationships between childhood psychosocial experiences (e.g. SES or adverse life events) and pubertal timing. Specifically, many of the latter authors were interested in how biological and social factors might interface to produce variable pubertal outcomes or they were specifically interested in antecedents of earlier menarche.

Most studies (*n =* 18) used age at menarche as the primary indicator for pubertal timing. In addition to age at menarche, some researchers elected to measure breast Tanner Stage (Graber et al., 1995), Peak Height Velocity (Mulligan et al., 1999) and a pre-pubertal development scale (Tan & Camras, 2015). Two studies did not use age at menarche at all but instead used breast and pubic Tanner Stage (Deardorff et al., 2011) or a combination of the Pubertal Development Scale, Tanner Stage and adrenal hormones at age 7 (Ellis & Essex, 2007). Energetic factors examined in relation to pubertal timing included height, weight, BMI, skinfold thickness, measures of body fat, thigh or arm circumference, breast development, stature, sitting height and subischial leg length (Table 2).

Psychosocial factors examined included measures of SES and environmental or familial stressors (e.g. adverse life events, familial conflict, parental stability, father absence, housing quality, sibling number and order, child behavioural problems and child psychological adjustment). At the very least, studies including SES typically measured it as a scale variable with education, income or both (Adair, 2001; Akther & Islam, 2012; Blell et al., 2008; Deardorff et al., 2011; Ellis & Essex, 2007; Kirchengast & Bauer, 2007; Moffitt et al., 1992; Mulligan et al., 1999; Nieczuja-Dwojacka et al., 2018; Orden et al., 2011; Rao et al., 1998; Tan & Camras, 2015; Windham et al., 2008). Other psychosocial factors used instead of or in addition to income–education SES markers included birth order (Akther & Islam, 2012; Deardorff et al., 2011; Krzyzanowska et al., 2016), recall of time lived in poverty (Reagan et al., 2012), family size or number of siblings (Akther & Islam, 2012; Krzyzanowska et al., 2016; Windham et al., 2008), housing conditions or crowding (Blell et al., 2008; Deardorff et al., 2011; Khan et al., 1996; Krzyzanowska et al., 2016) and social mobility (Krzyzanowska et al., 2016).

### Independent associations of childhood energetic factors and psychosocial stress with pubertal timing

In all studies critically reviewed, at least one measure indicative of greater pre-pubertal energetic status (e.g. larger relative body size) was significantly associated with accelerated pubertal timing. However, the specific energetic factors associated with pubertal timing varied across studies (Table 3). For example, Braithwaite et al. (2009) found that earlier age at menarche was associated with greater height, weight, percentage body fat, BMI, skinfolds and upper thigh circumference (see Table 3), but not with higher caloric intake or greater physical activity (Braithwaite et al., 2009). Null associations between pubertal timing and higher energy diets or physical activity were also observed in Nieczuja-Dwojacka et al. (2018) and Adair (2001).

Measures of psychosocial stress and their associations with pubertal timing were also varied. While some studies only included one measure such as SES, others included many measures (see Table 2 and 2). Eight studies (Adair, 2001; Deardorff et al., 2011; Ellis & Essex, 2007; Graber et al., 1995; Moffitt et al., 1992; Orden et al., 2011; Rao et al., 1998; Tan & Camras, 2015) found associations between earlier pubertal timing and all of their measured psychosocial variables, including advantaged SES, parental education, father absence, greater family conflict and low parental income. Graber et al. (1995) similarly found that all psychosocial variables (family conflict, less parental supportiveness, more depressive affect, and more internalizing and externalizing behaviours) were associated with earlier pubertal timing (Table 3).

Five studies (Braithwaite et al., 2009; Krzyzanowska et al., 2016; Nieczuja-Dwojacka et al., 2018; Reagan et al., 2012; Windham et al., 2008) found mixed associations between psychosocial exposures and pubertal timing. For example, in Windham et al. (2008), earlier age at menarche was associated with greater maternal education and income and having few or no siblings but not with family income at age 7 (Table 3). In contrast, in Nieczuja-Dwojacka et al. (2018) menarche was delayed with the presence of any siblings and there was no association between pubertal timing and maternal and paternal education or stress at home or school.

Reagan et al. (2012) found that white participants who had always lived in poverty experienced earlier menarche as compared with those who had never lived in poverty, but there was no association between poverty and menarche among Black participants. Conversely, Braithwaite et al. (2009) observed earlier age at menarche among lower income white and higher income Black participants (compared with higher income white and lower income Black participants), while age at menarche was not associated with family discord/environment or parental education across all participants. Kryzanowska et al. (2016) found that qualifying for free school meals and greater financial difficulties were associated with slower pubertal timing but found no association between pubertal timing and social mobility, social class or birth order. In both Reagan et al. (2012) and Kryzanowska et al. (2016) slower pubertal timing was associated with poverty, financial difficulties and qualifying for free school lunches. In seven studies (Akther & Islam, 2012; Blell et al., 2008; Khan et al., 1996; Kirchengast & Bauer, 2007; Kyweluk et al., 2018; Mulligan et al., 1999; Stark et al., 1989) none of the measured psychosocial variables (SES, birth order, adverse life events, parental income, number of siblings, sibling death and housing conditions) were associated with pubertal timing.

### What are the independent effects of psychosocial stress on pubertal timing when adjusting for energetic status?

All studies minimally included univariate tests or other independent assessments of both energetic and psychosocial factors on pubertal timing. However, only 11/20 studies evaluated energetic and psychosocial factors together in multivariate models. We summarized general findings across these studies, as methods were still too varied to allow for meta-analysis. We address only univariate vs. multivariate effects as a minimal essential step to consider confounding. Moreover, univariate and multivariate models have distinct explanatory power. Univariate models, when conducted as a first modelling step, help to identify dependent and independent relationships between psychosocial stressors and energetic factors on pubertal timing. However, one consequence of only presenting univariate results is heightened risk of unmeasured confounding, lack of interaction testing, and lack of precision. Thus, when psychosocial stress and energetic factors are measured together in a multivariate fashion, covariances can be decomposed more fully to unravel the biological meaning and plausibility of these relationships. Multivariate modelling conducted by authors is a first step towards necessary causal modelling, although causal inference models were rarely used in the studies reviewed.

In 9/11 multivariate models (Adair, 2001; Akther & Islam, 2012; Khan et al., 1996; Krzyzanowska et al., 2016; Kyweluk et al., 2018; Orden et al., 2011; Reagan et al., 2012; Windham et al., 2008) energetic factors were significantly associated with pubertal timing when also controlling for psychosocial variables. Two studies used mediation models to test whether psychosocial stress was indirectly associated with pubertal timing via influence on childhood growth or body composition – reasoning, for example, that child behavioural problems are associated with negative psychological affect, which may promote increased body fat through altered eating, metabolism or physical activity (Ellis & Essex, 2007; Moffitt et al., 1992). Moffit et al. (1992) first tested for main effects of father absence, child behavioural problems at age 7, family conflict and weight at age 9 and found that all but child behavioural problems were significantly associated with earlier age at menarche. In additive models, menarche was predicted to be earlier among individuals with two or three of these risk factors and much earlier in individuals with all four risk factors. However, father absence appeared to be the main driver of these associations across all cumulative risk combinations. In mediation models, behavioural problems and family stress (i.e. father absence/family conflict) were not indirectly associated with menarche via body weight at age 9. Ellis and Essex (2007) similarly hypothesized that BMI may mediate the relationship between marital conflict/depression and pubertal development in the fifth grade, after finding that BMI, maternal age at menarche, maternal parental supportiveness and SES each independently associated with pubertal timing. They also did not find evidence of mediation by BMI. In contrast, they did find a large direct effect of BMI on pubertal development in the fifth grade and a slightly smaller indirect effect of marital conflict/depression on pubertal development (Table 4).

In 8/11 multivariate models (Adair, 2001; Akther & Islam, 2012; Ellis & Essex, 2007; Krzyzanowska et al., 2016; Moffitt et al., 1992; Orden et al., 2011; Tan & Camras, 2015; Windham et al., 2008), at least one psychosocial factor was significantly associated with pubertal timing when controlling for measured energetic factors. In the remaining three (Khan et al., 1996; Kyweluk et al., 2018; Reagan et al., 2012), none of the psychosocial variables measured were significantly associated with pubertal timing when controlling for energetic factors. For example, in a multivariate model in Kyweluk et al. (2018), age at menarche was inversely related to BMI and caloric intake for body weight but was not associated with sibling death or maternal or paternal absence.

## Discussion

In our selection process, we identified only 20 studies that examined the influence of both childhood energetic factors and psychosocial stress on pubertal timing, and only 11 of these accounted for both in multivariate models to address potential confounding (Table 4). Across all selected studies, energetic factors were more consistently associated with pubertal timing than were psychosocial factors, with measures indicative of rapid childhood growth generally predicting earlier pubertal timing. Across the 20 studies, eight found associations between pubertal timing and all measured psychosocial variables, five found mixed results and seven found no associations. In multivariate models in which both energetic and psychosocial factors were significantly associated with pubertal timing, we observed no consistent patterns of *stronger* effects for energetic vs. psychosocial variables.

It is important to note that both insults from psychosocial stressors and energetic resources vary at higher-order population levels and more local, granular levels. In this review, most studies (*n =* 13) were conducted in environments such as the USA or the UK where greater psychosocial stress and obesity rates may be higher among impoverished or disadvantaged groups owing to structural inequalities and highly sedentary lifestyles (El-Sayed et al., 2012; Min et al., 2018). However, in other contexts reviewed here, such as Guatemala, impacts on pubertal growth and timing may play out differently in the context of dual burdens of rising overweight and obesity (overnutrition), *and* undernutrition (Doak et al., 2016). In this way, it is reasonable that there may be some ascertainment bias in our selected sample of studies.

To highlight the complexity and importance of considering population contexts, we show how psychosocial and energetic factors may result in diverse outcomes based on varying environmental contexts in Table 5. In environments characterized by high psychosocial stress and low resources it is expected that puberty will be delayed from an energetic perspective. Psychosocial Acceleration Theory might suggest pubertal timing is accelerated in a high stress context, whereas Suppression Theory may suggest it is delayed. In a Low Stress/Low Resource context, we expect energetic factors and psychosocial stress to delay puberty, although in the modern context, we are not sure Low Stress/Low Resource environments exist (thus marked by a question mark). In High Stress/High Resource contexts, both energetic and psychosocial factors are expected to accelerate pubertal timing, especially when poverty is associated with both higher BMI and high psychosocial stress at the population level. Lastly, in a Low Stress/High Resource setting, we might expect that advantaged wealth may relate to higher BMI but may also be protective towards psychosocial stress and thus we expect opposite effects when considering energetic or psychosocial impacts on pubertal timing. Moreover, we underscore that those latent variables such as energetic sufficiency or obesity are not often modelled and when they are modelled – for example as SES – impacts on pubertal timing may be variable (see Table 5). Future research can more definitively assess the influence of psychosocial stress on pubertal timing by incorporating cross-group comparisons drawn from populations in which obesity is and is not correlated with greater wealth and socioeconomic status. Opportunities for such cross-population comparisons may become increasingly limited as obesity risks are trending upwards among marginalized populations in low- and middle-income countries as well (Masood & Reidpath, 2017).

**Table 5.** Summary of potential energetic and psychosocial impacts on pubertal timing under varying resource and stress environments

	High stress/low resource	Low stress/low resource	High stress/high resource	Low stress/high resource
Energetic	+	+	−	−
Psychosocial	+ or −	+	−	+
Potential population contexts	Disadvantaged status is associated with greater stress and undernutrition High social inequalities	More advantaged status is associated with lower stress and undernutrition Uncertain if these circumstances ever co-exist (?)	Disadvantaged status is associated with greater stress and overnutrition High social inequalities	More advantaged status is associated with lower stress and overnutrition Historical local diets (pre-Nutritional Transition)

−, Earlier puberty; +, delayed puberty; low/high resource, relative energetic scarcity or abundance.

Our review demonstrates the need for greater theoretical and mechanistic clarity as to how and why childhood psychosocial stress may affect the plasticity of pubertal timing (see Figures 1–3). Only nine studies we reviewed provided at least a partial explanation or discussion of why psychosocial factors were expected to influence pubertal timing (Braithwaite et al., 2009; Deardorff et al., 2011; Ellis & Essex, 2007; Graber et al., 1995; Khan et al., 1996; Kirchengast & Bauer, 2007; Kyweluk et al., 2018; Moffitt et al., 1992; Stark et al., 1989). Of these, five referred specifically to Acceleration Theory or the potential for evolutionary life history strategies to calibrate pubertal timing based on psychosocial stress experienced in childhood (Deardorff et al., 2011; Ellis & Essex, 2007; Graber et al., 1995; Kyweluk et al., 2018; Moffitt et al., 1992). While we do not view SES as a psychosocial variable *per se*, some authors perceived it as such. For example, 4/9 studies provided discussion about the ways SES or psychosocial stress may be felt through differential social, nutritional or economic status (Braithwaite et al., 2009; Khan et al., 1996; Kirchengast & Bauer, 2007; Stark et al., 1989). However, explicit pathways were not explored to plausibly connect psychosocial stress signals to pubertal outcomes. As we highlight in Table 5, we might expect contradictory pubertal outcomes depending on population context and the extent to which SES or poverty are considered.

Moreover, if SES continues to be used as an indicator of stress, it would be worthwhile to disentangle stressful experiences related to social inequalities from direct effects of resource availability. One such way to do this would be to integrate both objective and subjective measures of SES. For example, García et al. (2017) highlight how direct (e.g. resource availability) and indirect (e.g. perceived unequal distribution of resources) correlates of SES may differentially impact HPA activation (García et al., 2017). Taking it a step further, testing objective and subjective SES in relation to HPA reactivity and HPG hormones prospectively up until puberty may help distinguish which dimensions of SES (e.g. perceived distribution of resources, social status stress, or crude resource availability) are signals of psychosocial stress and how they may influence pubertal timing via acceleration or suppression of the HPG.

While about half of our selected studies explicitly outlined mechanisms or at least acknowledged their predictive potential, there should be more attention paid to further explain biologically meaningful differences among proxy measures of psychosocial stress such as adverse childhood events or familial conflict. For example, B. Bogin (2021a, b) proposes two specific pathways by which adverse experiences can impact growth, such as upregulation of glucocorticoids and calcitonin via chronic stress and downregulation of growth hormone/IGF-1 with downstream neuroendocrine effects (B. Bogin, 2021a, b). Thus, it would be particularly beneficial given the heterogeneity of stressors examined here, to think through which factors are more reliably or more likely to promote these biological processes. As an example, the extent to which studied factors such as household crowding, birth order or parental report of marital problems consistently signal psychosocial stress is unclear in our view. The biological plausibility that these factors reliably impact childhood growth or promote acute or chronic biological stress responses is not well tested to our knowledge.

Likewise, for many stress indicators there is the issue of construct validity. For example, psychosocial stress variables such as lifetime stress, adverse stress scales or binary variables related to school or home stress are unlikely to quantify individual perception or appraisal of psychosocial stress or continuity of stress effects. Still, regardless of whether observed factors signal psychosocial stress, latency between childhood exposures and pubertal outcomes introduces potential ‘noise’, especially when underlying biological mechanisms or upstream programming (e.g. epigenetic programming) go unmeasured. At the same time, some psychosocial variables studied here, such as sibling loss or adverse family experiences do attempt to capture environmental stressors which may have an impact on demographic patterning. Future researchers may consider implementing multiple types of study instruments to achieve more comprehensive measurement, innovate methods to study psychosocial stress signals and their subjective and biological impact, and use more complex statistical approaches such as causal inference and structural equation modelling to capture latent vs. observed psychosocial impacts on pubertal timing. Even more important, is that future research will be bolstered by considering socioecological contexts (see Table 5) and integrating these into study designs and theoretical predictions to merge the apparent gap between evolutionary theory and biological plausibility.

Belsky, Chisholm and others have long acknowledged the importance of mechanisms linking psychosocial stress and earlier menarcheal timing, such as HPA axis ‘dysregulation’ (Belsky et al., 2007; Chisholm, Burbank, et al., 2005). Of note, we excluded Belsky et al. (2007) during our methods screening. This paper examined family factors associated with earlier puberty but did not mention energetic factors as variables of interest in the methods nor include any anthropometric measures as covariates in models. However preliminary analyses reported in the results did note that both height and weight in fourth grade were moderately inversely correlated with pubertal onset. Only two studies in our review attempted (Ellis & Essex, 2007; Moffitt et al., 1992) to account for confounding between psychosocial variables and relative adiposity through mediation analysis.

In addition, we did not formally assess risk of bias. However, we qualitatively assessed whether primary aims of the selected studies may have influenced their results. Of seven studies designed to primarily test the influence of energetic factors on pubertal timing, all of them found evidence for those predicted relationships. Four of those studies also found associations between psychosocial variables and pubertal timing, the other three did not. Similarly, all seven studies designed to explicitly test the influence of psychosocial factors on pubertal timing found evidence for at least one the predicted relationships, and all of them found associations with energetic factors. For the remaining six studies explicitly designed to test both psychosocial and energetic factors, all found an association between energetic factors and pubertal timing, but four of the six found no association with psychosocial factors.

Overall, our review highlights that in the pubertal context, few studies simultaneously account for factors indicative of childhood energetic status and psychosocial stress on pubertal timing. We have highlighted some key future research directions and we have helped synthesize and update prior critiques of evolutionary hypotheses about variable pubertal timing. Critically, we also advance ideas to bring together evolutionary hypotheses explaining variable pubertal timing and knowledge about human biological variation in adolescence. Taken together, we hope to propel fruitful research directions regarding psychosocial stress exposures and energetic status. A strength of this review is that it includes only studies that measured energetic factors prior to menarche – either prospectively or using status quo methods with a sufficiently large proportion of premenarcheal participants – which limits confounding in results owing to continued post-menarcheal increases in stature and body fat. However, we included studies that measured pubertal outcomes and psychosocial factors retrospectively, which may be biased by recall error. Lastly, the studies presented here focused on human female pubertal timing, and thus do not represent all potential variability among sexes (e.g. intersex, male).

## Research transparency and reproducibility

Our systematized review protocol and search strings can be found on Open Science Framework: https://osf.io/nckrh/
